# High Efficiency Membranes Based on PTMSP and Hyper-Crosslinked Polystyrene for Toxic Volatile Compounds Removal from Wastewater

**DOI:** 10.3390/polym14142944

**Published:** 2022-07-20

**Authors:** Georgy Golubev, Stepan Sokolov, Tatyana Rokhmanka, Sergey Makaev, Ilya Borisov, Svetlana Khashirova, Alexey Volkov

**Affiliations:** 1 A.V. Topchiev Institute of Petrochemical Synthesis RAS, 29 Leninsky prospekt, 119991 Moscow, Russia; sokolovste@ips.ac.ru (S.S.); tatyana_rokhmanka@mail.ru (T.R.); makaev@ips.ac.ru (S.M.); boril@ips.ac.ru (I.B.); avolkov@ips.ac.ru (A.V.); 2 Department of Organic Chemistry and Macromolecular Compounds, Kabardino-Balkar State University named after H.M. Berbekov, 173 Chernyshevsky St., 360004 Nalchik, Kabardino-Balkarian Republic, Russia; sveta_daova@mail.ru

**Keywords:** aromatic hydrocarbons, benzene, toluene, o-xylene, BTX, removal, PTMSP, polystyrene, mixed matrix membranes, pervaporation

## Abstract

For the first time, membranes based on poly(1-trimethylsilyl-1-propyne) (PTMSP) with 5–50 wt% loading of hyper-crosslinked polystyrene sorbent particles (HCPS) were obtained; the membranes were investigated for the problem of effective removal of volatile organic compounds from aqueous solutions using vacuum pervaporation. The industrial HCPS sorbent Purolite Macronet™ MN200 was chosen due to its high sorption capacity for organic solvents. It has been found that the membranes are asymmetric when HCPS content is higher than 30 wt%; scanning electron microscopy of the cross-sections the membranes demonstrate that they have a clearly defined thin layer, consisting mainly of PTMSP, and a thick porous layer, consisting mainly of HCPS. The transport and separation characteristics of PTMSP membranes with different HCPS loading were studied during the pervaporation separation of binary and multicomponent mixtures of water with benzene, toluene and xylene. It was shown that the addition of HCPS up to 30 wt% not only increases the permeate fluxes by 4–7 times, but at the same time leads to 1.5–2 fold increase in the separation factor. It was possible to obtain separation factors exceeding 1000 for all studied mixtures at high permeate fluxes (0.5–1 kg/m^2^∙h) in pervaporation separation of binary solutions.

## 1. Introduction

One of the main pollutants of groundwater and industrial wastewater from oil refineries and chemical plants are volatile organic compounds (VOCs), mainly aromatic hydrocarbons (benzene, toluene, xylenes (BTX)) [1,2]. BTX are well known for their chronic toxicity and mutagenic potential. For example, benzene is one of the most toxic compounds and causes hematological disorders, including leukemia, even at low concentrations [3]. Due to the low solubility of these compounds in water, the concentrations of VOCs in water are too small for economically viable removal using traditional separation—distillation technology.

Instead, such processes as filtration, biological purification, membrane separation, and adsorption are used for wastewater and groundwater treatment [4,5,6]. Among them, membrane separation technology has great advantages in terms of potential savings in energy costs [6]. At petrochemical plants, a large amount of low-potential heat is always available; it can be used to create the driving force of separation processes for the release of VOCs from aqueous solutions by membrane methods. The most effective of them is pervaporation. Pervaporation is a process of separation of liquid mixtures using dense membranes, where permeate is removed in the form of vapor [7]. Despite a significant number of works on the removal of volatile organic compounds (VOCs) from aqueous solutions by pervaporation, the range of membrane materials used for this task is quite narrow. The most common of these materials are: hydrophobic silicalites [8], silicone rubbers (PDMS, POMS) [6,9,10,11,12], as well as highly permeable polymer glasses (PTMSP, PIM-1) [13,14,15]. The vast majority of scientific papers on the removal of VOCs from water by the pervaporation are devoted to the study of membranes based on polysiloxanes, which often do not provide sufficiently high selectivity [14,16]. Hydrophobic glassy polymers with a large proportion of free volume, in particular poly(1-trimethylsilyl-1-propyne) (PTMSP), are considered promising pervaporation membrane and sorption materials for separating liquids [17,18,19,20]. The increased interest in these polymers is due to their outstanding properties, in particular, high permeability due to a large proportion of free volume. However, a significant disadvantage of glassy polymers is physical aging over time and, as a consequence, a significant decrease in permeability coefficients [21,22]. This creates a serious problem for the commercial use of these polymers.

One of the most common ways to reduce the effect of aging, as well as to increase the selectivity of the membrane material, is the introduction of a filler phase to obtain the so-called mixed matrix membranes (MMM) [23,24,25]. Zeolites [26], metal-organic frameworks [27], activated carbon [28], polyhydroxyfullerenes [29], graphene [30], porous aromatic framework structures (PAF) [31], hyper-crosslinked polystyrene (HCPS) [32] are used as such fillers. These additives increase the total free volume of the material, which in turn leads to an increase in the diffusion coefficients and/or solubility of the target components of the separated mixture. As a result, the permeability and selectivity increases in MMM.

It has been demonstrated, for example, by pervaporation mixed matrix membranes for the removal of volatile organic compounds from wastewater. MMM filled with molecular sieves, zeolites, and activated carbon adsorbents demonstrate increased selectivity and permeability in comparison with unmodified membranes [26,28,33,34]. For a carbon-modified PDMS membrane, toluene removal after one test cycle was about 80% [34]. Modification of silicone rubbers with hydrophobic zeolites [26] and microporous metal-organic frameworks [27,35] leads to an increase in the tortuosity of the diffusion path of water, which is expressed in an increase in organic components flux through the membrane and increase in the organic/water separation factor. The addition of polyoxometalates and carbon-based nanomaterials leads to an increase in the separation characteristics of the membranes [36,37,38]. Loading with fullerenols (content in the membrane is 5 wt%) makes it possible to increase the permeate flux, selectivity and mechanical properties in the pervaporation separation of water-alcohol mixtures, compared to unmodified membranes [29]. The loading of PTMSP with up to 3 wt% HCPS allowed the separation factor to increase in comparison with homogeneous membranes during thermopervaporation separation of a binary butanol/water mixture [32].

Choosing the optimal additive/polymer pair for the task of the removal of VOCs from wastewater, using benzene, toluene and xylene as the examples, as well as production of MMM based on the found composition, is a serious fundamental task. Hyper-crosslinked polystyrenes deserve special attention due to their high adsorption activity, simple and cheap method of production, and the ability to regenerate by sufficiently economical methods [39]. HCPS were first obtained by Davankov, Rogozhin and Tsyurupa in the 1970s and represent a class of nanoporous materials with a wide range of practical and potential applications [39]. Simple crosslinking of aromatic monomers by the Friedel–Crafts reaction is used to produce HCPS. Variation in the synthesis technique allows the obtaining samples of HCPS with mono- and bimodal pore size distribution. Systematic studies of these sorbents by many methods, including positron annihilation [40], confirmed that such materials have an open microporous structure and a high fraction of free volume, about 30% [41]. Due to the rigid open structure of the hyper-crosslinked mesh, the material is an excellent sorbent and, as such, has found wide application in industry, environmental protection, analytical chemistry and even as a unique hemosorbent in medicine [39]. Earlier it was shown that the industrial sample of HCPS (Purolite MN-202) was effectively used as an adsorbent for the separation of benzene, toluene and o-xylene. Compared to industrial adsorbents, such as activated carbon (Carbon CD500) and bentonite clay (Claytone-40), HCPS demonstrated the maximum absorption capacity for benzene, toluene and o-xylene (0.8 ± 0.1; 0.70 ± 0.08 and 0.63 ± 0.06 mmol/g at 26 °C), which indicates the prospects of these materials for separation of BTX aqueous solutions [42]. Significant intensification of the process of VOCs removal from water can be achieved by creating membranes based on PTMSP and highly effective sorbents, therefore combining the advantages of sorption and pervaporation separation methods.

In this article, fundamentally new mixed matrix membranes based on commercial PTMSP (Gelest, Inc., Morrisville, PA, USA) and HCPS (Purolite Ltd., Macronet™ MN200, Llantrisant, UK) with a content of up to 50 wt% were obtained and investigated for the first time; the sorption and transport function are performed by finely dispersed HCPS, while the mechanical and film-forming properties are provided by PTSMP. The transport and separation properties of PTMSP-HCPS membranes were studied during vacuum pervaporation separation of model binary mixtures of benzene–water, toluene–water, xylene–water and multicomponent model mixture benzene–toluene–xylene–water.

## 2. Materials and Methods

### 2.1. Materials and Reagents

PTMSP (SSP-070, lot 9D-35578, MW 250 × 10^3^ g/mol [43]) was purchased from Gelest, Inc. (Morrisville, PA, USA). Macronet ™ MN200 commercial sorbent (batch: 113X/18/15) was purchased from Purolite Ltd. Chloroform (chemical grade, CHIMMED, Moscow, Russia) was taken without further purification.

### 2.2. Preparation of PTMSP and PTMSP/HCPS Membranes

A total of 1.5 wt% PTMSP solution in chloroform was used for membrane preparation. Dense PTMSP membranes were fabricated by casting PTMSP-chloroform solution on cellophane and subsequent drying for 200 h under ambient conditions at room temperature. The initial film diameter was 7.5 cm. The thickness was in the range of 35–45 μm. The thickness of the membranes was measured using a Mitutoyo^®^ 293 Digimatic QuickMike electron micrometer (Mitutoyo Corporation, Kawasaki, Kanagawa, Japan) with an accuracy of ±1 μm [44].

To produce MMM, an industrial biporous (macro- and micropores) sorbent based on hyper-crosslinked polystyrene Macronet™ MN200 (Purolite Ltd., Llantrisant, UK) with specific surface area 1100 m^2^/g and density 0.48 g/cm^3^ was used as a filler [45]. The initial size of MN200 spherical pellets varied in the range of 300–1200 µm. Polymer dispersion was obtained by two-stage grinding of sorbent granules in a swollen state, at the first stage in a vibrating ball mill and an ultrasonic sonicator at the second. The dispersion of sorbents in chloroform was stirred for 24 h at room temperature to obtain swollen granules. Then the sorbent granules were ground in a swollen state in a laboratory ball mill IBMT-30 (HT MACHINERY Co., Ltd., Harbin, China). After that, a dispersion of HCPS particles in chloroform was intensively treated for 5 min using an ultrasonic sonicator UZD2-0.1/22 (UZTO LLC, St. Petersburg, Russia) at 22 kHz operating frequency with creation of ultrasonic cavitation. The average particle size of HCPS used as a membrane filler was close to 650 nm.

The PTMSP/HCPS casting suspensions with different filler content (0, 5.0, 10, 30 and 50 wt%) were prepared by mixing two mixtures containing 1.5 wt% and 1.5 wt% of PTMSP and HCPS in chloroform, respectively. Before mixing with polymer solutions, the HCPS suspension was placed in the ultrasonic bath for 15 min. Then, prior to membrane casting, the PTMSP/HCPS suspensions were stirred using magnetic bar for 35 min and were placed into the ultrasonic bath for another 15 min. The PTMSP/HCPS suspensions were cast onto cellophane, blanketed by a glass plate for slow evaporation of the solvent in ambient conditions (about 200 h) and dried to a constant weight. The resulting thickness of the PTMSP/HCPS membranes was in the range of 45–70 µm.

### 2.3. Particle Size Distribution

The size distributions of HCPS particles obtained after grinding the initial commercial MN200 samples were determined by dynamic light scattering on a Malvern Zetasizer Nano analyzer (Malvern Panalytical Ltd., Malvern, Worcestershire, UK) [46]. The test samples were prepared by suspending the particles in chloroform (0.06 g of a sample in 10 mL of solvent).

### 2.4. Membrane Characterization

Scanning electron microscopy (SEM) was used to characterize the structure and morphology of the membranes. SEM was carried out on a Thermo Fisher Phenom XL G2 Desktop SEM (Thermo Scientific, Waltham, MA, USA). Cross-sections of the membranes were obtained by fracturing in liquid nitrogen after preliminary impregnation of the specimens in isopropanol. A thin (5–10 nm) gold layer was deposited on the prepared samples in a vacuum chamber (~0.01 mbar) using a desktop magnetron sputter “Cressington 108 auto Sputter Coater” (Cressington Scientific Instruments Ltd., Watford, UK). The accelerating voltage during images acquisition was 15 kV. Further image analysis and determination of the selective layer thickness was carried out using the Gwyddion software ver. 2.53 (David Nečas (Brno University of Technology, Czech Republic) and Petr Klapetek (Czech Metrology Institute, Czech Republic).

### 2.5. Contact Angle Measurement

The measurements of contact wetting angles were performed by the standard method of a lying drop using a LK-1 goniometer manufactured by RPC OpenScience Ltd. (Krasnogorsk, Russia). The measurements were carried out on both surfaces of hybrid membranes with different filler content of HCPS particles. Data acquisition and subsequent digital processing of droplet images for the direct calculation of angles using the Young–Laplace equation was carried out using DropShape software. The measurement error was ±2°. The experiments were carried out at the ambient temperature of 23 ± 2 °C.

### 2.6. Vacuum Pervaporation

**Figure 1:**
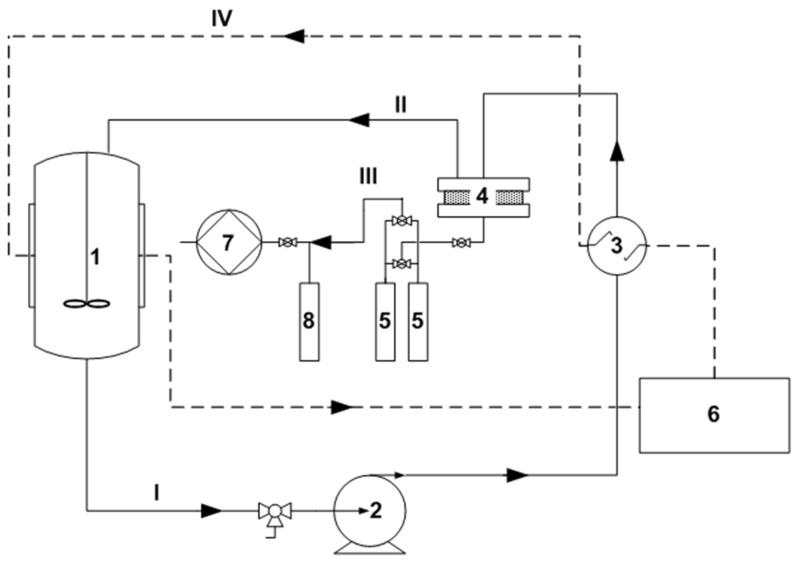
Scheme of the vacuum pervaporation setup: 1—a container with a mixing device; 2—a gear pump; 3—a heat exchanger; 4—a membrane module; 5—traps for collecting permeate, placed in Dewar vessels with liquid nitrogen; 6—thermostat; 7—vacuum pump; 8—safety trap; I—initial separable mixture; II—retentate; III—permeate; IV—coolant.

Pervaporation experiments were carried out on the setup shown in Figure 1.

The initial feed mixture was poured into a 2 L container (1). The mixture was heated in the heat exchanger (3) and fed into the membrane module (4) in a circulating mode using an Ismatec (Switzerland) gear pump (2). The volumetric flow rate of the feed mixture was 350 mL/min. The effective membrane area was 13.9 cm^2^. Permeate vapors were condensed in glass traps placed in Dewar vessels with liquid nitrogen (−196 °C) (5). The continuous operation of the installation throughout the experiment was ensured by the presence of two traps, working in parallel. The temperature of the feed mixture was maintained with an accuracy of ±0.1 °C using a LOIP LT-100 (Russia) liquid thermostat (6). The driving force of the mass transfer process (a pressure of ~0.05 mbar in the submembrane space) was created by and maintained with an Ebara PDV-250 (Japan) vacuum pump (7). A safety trap (8) was used to prevent permeate vapor from entering the vacuum pump.

The study of pervaporation of BTX-water solutions was carried out at a temperature of 30 (±0.1) °C. The initial solutions were prepared from benzene, toluene, o-xylene (laboratory grade, chemically pure) and distilled water by gravimetric method. The initial concentrations of the target components in binary solutions were as close as possible to their solubility in water, for example, for benzene, the maximum solubility in water at 20 °C is 0.18 wt% [47], for toluene is 0.052 wt% [48], and for o-xylene is 0.02 wt% [49]. For a multicomponent solution of benzene-toluene-o-xylene-water, the concentrations of the components were 0.01-0.01-0.01-99.97 wt%. This composition of the multicomponent mixture of BTX is the most common in the literature and is close to the content of volatile organic compounds in the wastewater of petrochemical industries [50]. The exact concentrations of BTX in the feed mixture and permeate were determined by gas chromatography on a Kristallux-4000M gas chromatograph (“Meta-chrome”) equipped with a flame-ionization detector. Chromatography parameters: evaporator temperature was 200 °C, column temperature was 120 °C, detector temperature was 150 °C. The measurements were carried out using a Phenomenex Zebron ZB-FFAP capillary column (length 50 m, diameter 0.32 mm, film thickness 0.50 µm), phase: nitroterephthalic acid modified polyethylene glycol. Water was added before the analysis to homogenize the permeate samples, which contained a two-phase system.

Flux was estimated by weighing permeate collected over a given period of time. Total permeate flux was calculated as:

(1) $$J_{t}{}^{{}_{}}=\frac{\Delta m}{S\cdot \Delta t}$$

where Δ*m* is the weight of the permeate (kg), which passed through the membrane with area *S* (m^2^) within a time Δ*t* (h).

The separation factor was determined by the formula (2):

(2) $$\alpha =\frac{y_{o}\cdot x_{w}}{y_{w}\cdot x_{o}}$$

where *x_o_* and *x_w_* are the mass fractions of the organic component and water in the separated mixture, and *y_o_* and *y_w_* are the mass fractions of the organic component and water in the permeate. The separation factors, in the case of a four-component mixture of benzene-toluene-o-xylene-water, were calculated as for the binary systems benzene–water, toluene–water, o-xylene–water.

The mass fluxes of components in permeate were determined as:

(3) $$J_{i}=J_{t}\cdot y_{o}$$

The efficiency of the pervaporation process was characterized by the pervaporation separation index (PSI), which takes into account the dependence of two factors—the permeate flux and the separation factor:

(4) $$PSI=J_{t}\cdot \left(\alpha -1\right)$$

The corrected sample standard deviation from the mean value (*Ȳ*) in the given series of measurements was taken as the measurement error (Δ*Y*):

(5) $$\Delta Y=\sqrt{\overset{n}{\underset{k=1}{\sum }}\frac{{\left(Y_{n}-\bar{Y}\right)}^{2}}{n-1}}$$

where *Y* is the calculated value, *n* is the number of measured values.

## 3. Results

### 3.1. PTMSP/HCPS Membranes

**Figure 2:**
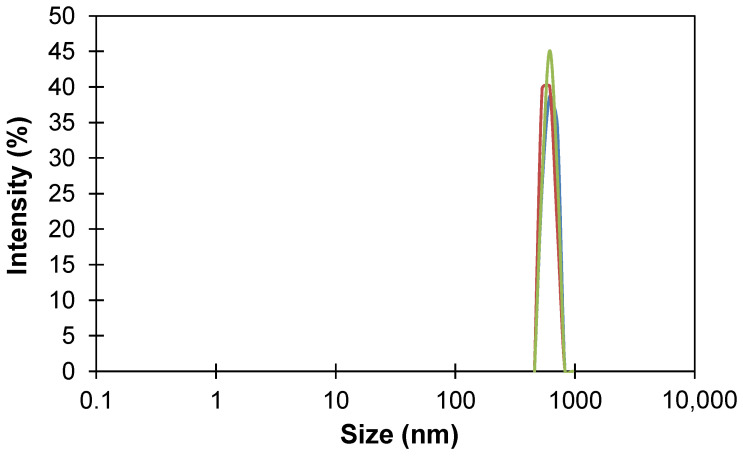
Size distribution of HCPS MN200 in chloroform.

Hyper-crosslinked polymers were first produced by Davankov, Rogozhin, and Tsyurupa and are now produced in volume by Purolite Corporation. The commercial hyper-crosslinked polystyrene sorbent Macronet^TM^ MN200 was chosen as a modifying additive, since it is the most used sorbent of the Macronet^TM^ family for the removal of volatile organic compounds from aqueous solutions and it also exhibits a high surface area (1100 m^2^/g) [45]. The initial size of MN200 spherical pellets varied in the range of 300–1200 µm, therefore, sorbent granules were crushed in a swollen state in a vibrating ball mill at the first stage and an ultrasonic sonicator at the second stage to produce MMM. Figure 2 shows the typical particle-size distribution of milled MN200 particles in the dispersion in chloroform. The average particle size was determined by the maximum of intensity, so the HCPS particle size after milling was 650 nm.

**Figure 3:**
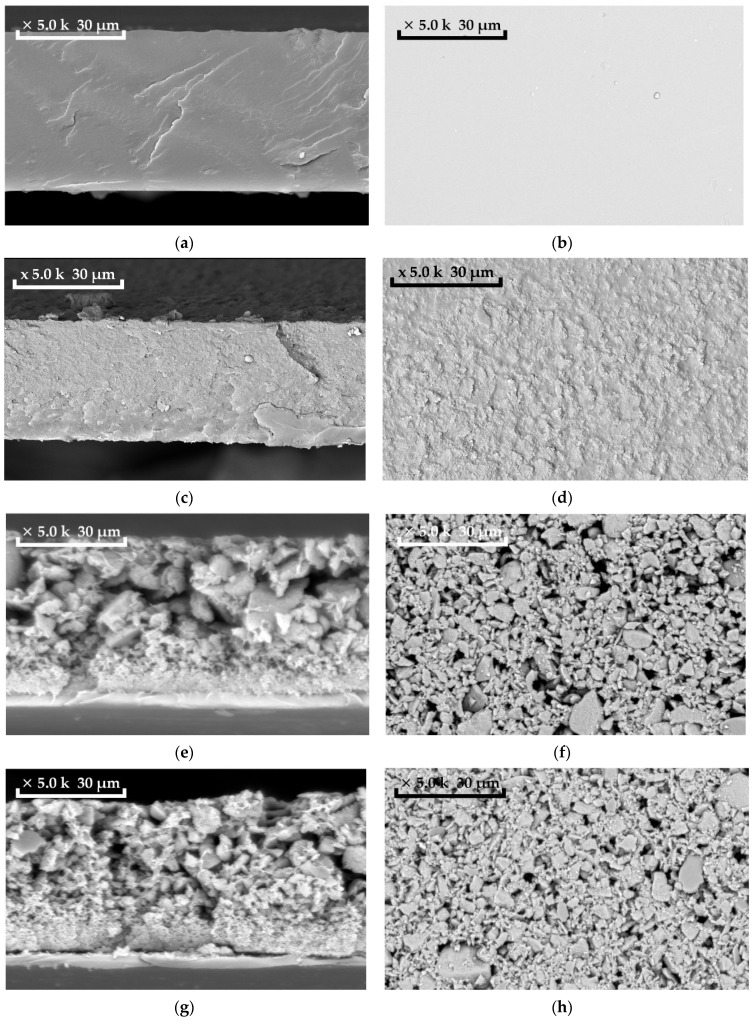
SEM images of membranes: (**a**) cross-section of PTMSP membrane, magnification 5000×; (**b**) top view of PTMSP membrane, magnification 5000×; (**c**) cross-section of membrane with 10 wt% HCPS content in PTMSP, magnification 5000×; (**d**) top view of the membrane with 10 wt% HCPS content in PTMSP, magnification 5000×; (**e**) cross-section of the membrane with 30 wt% HCPS content in PTMSP, magnification 5000×; (**f**) top view of the membrane with 30 wt% HCPS content in PTMSP, magnification 5000×; (**g**) cross-section of the membrane with 50 wt% HCPS content in PTMSP, magnification 5000×; (**h**) top view of the membrane with 50 wt% HCPS content in PTMSP, magnification 5000×.

**Figure 4:**
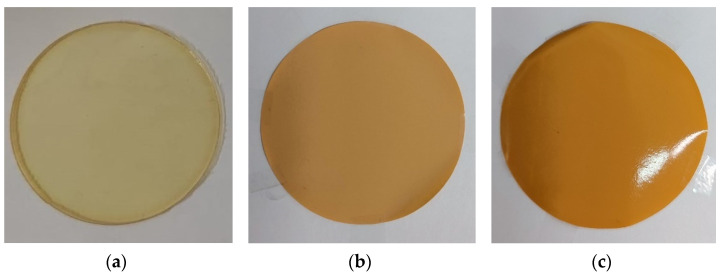
PTMSP (**a**), PTMSP/HCPS 50 wt%—surface principally consisting of HCPS (**b**), PTMSP/HCPS 50 wt%—surface principally consisting of PTMSP (**c**).

By mixing PTMSP solution and HCPS suspension in chloroform, hybrid membranes with variable content of HCPS particles (from 5 to 50 wt%) were produced. In previous studies, PTMSP membranes with a maximum load of 10 wt% HCPS were obtained [43]. The morphology of MMM reflects the structural features of the polymer matrix system with impregnated particles and is one of the main factors determining the physical properties of such hybrid membranes. The distribution of HCPS particles on the surface and in the volume of the membranes was studied by scanning electron microscopy (SEM) (Figure 3). As can be seen from Figure 3a,b, virgin dense PTMSP membrane had a smooth homogeneous surface. When adding 10 wt% of HCPS, a loose structure of the polymer matrix of PTMSP is formed (Figure 3c,d). SEM of the cross-section of PTMSP/HCPS 90/10 wt% membrane (Figure 3c) demonstrates that HCPS particles have a fairly uniform distribution in the PTMSP matrix; however, an increased content of HCPS particles is observed on one of the membrane surfaces. Most likely, this happens during the membrane forming process. The movement of HCPS particles to the membrane surface occurs due to the lower density of HCPS (0.5 g/cm^3^) than that of PTMSP (0.75–0.79 g/cm^3^), as a result of which, HCPS particles float to the surface during the membrane production process. This fact is more obvious in Figure 3e,g, where HCPS content in PTMSP is 30 and 50 wt%, correspondingly. The membranes are clearly asymmetric, and the cross-sections (Figure 3e,g) of the membranes are similar to composite membranes (a clearly defined thin layer consisting mainly of PTMSP and a thick porous layer consisting mainly of HCPS). Figure 4 shows SEM photos of membranes from PTMSP (a) and PTMSP/HCPS 50 wt% (b,c). The membrane surface is rough and matte on the side principally containing HCPS (Figure 4b), and smooth and glossy on the reverse side, principally containing PTMSP (Figure 4c). The layer principally containing PTMSP, 3–5 µm thick, also contains HCPS particles. It is worth noting that the layer, principally consisting of HCPS, has a thickness of 50–60 µm and is firmly held in the membrane due to PTMSP acting as a binding component. Most likely, the mechanical properties of these membranes are mainly provided by a thin layer of PTMSP. As can be seen, HCPS particles agglomerate during the drying process to form individual clusters up to 15 µm in size (Figure 3e–h). Further increase in HCPS content to 60 wt% resulted in the formation of brittle membrane, which ultimately collapsed.

**Figure 5:**
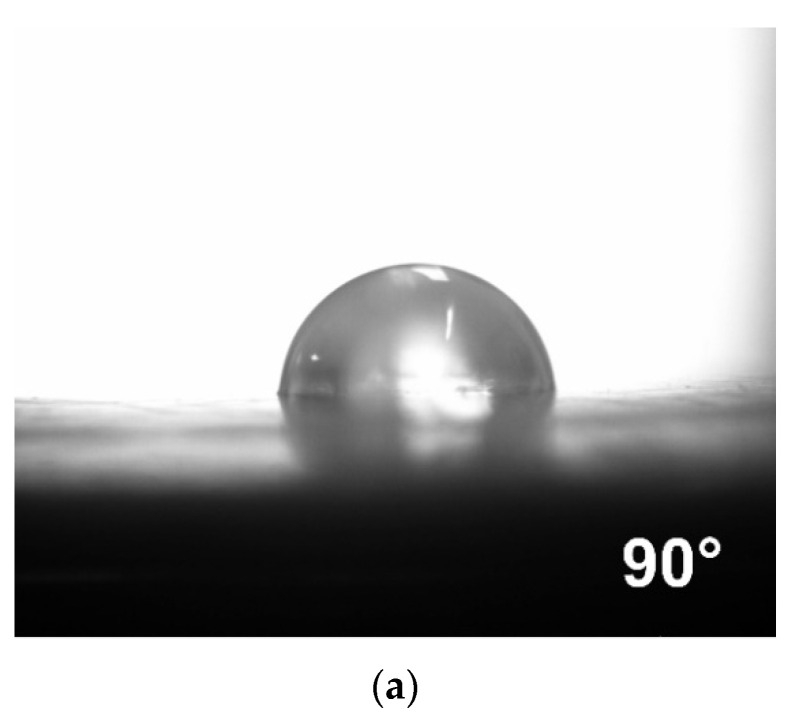

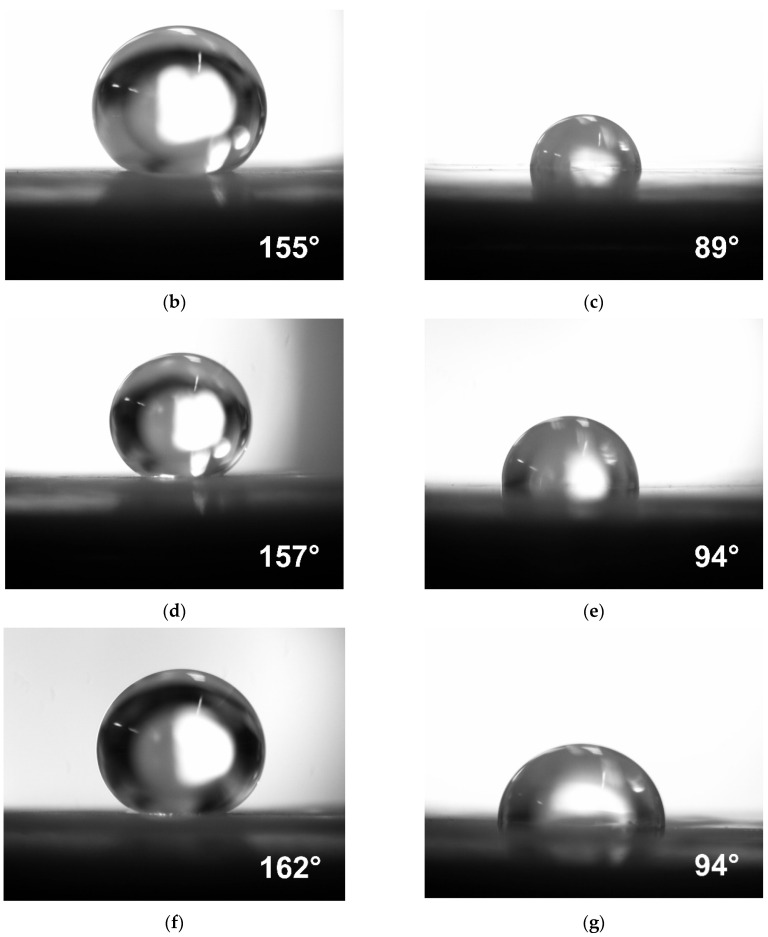
Measurements of the water contact angle of the membrane surface: (**a**) PTMSP membrane; (**b**) PTMSP/HCPS 90/10 wt% from the surface principally consisting of HCPS; (**c**) PTMSP/HCPS 90/10 wt% from the surface principally consisting of PTMSP; (**d**) PTMSP/HCPS 70/30 wt% from the surface principally consisting of HCPS; (**e**) PTMSP/HCPS 70/30 wt% from the surface principally consisting of PTMSP; (**f**) PTMSP/HCPS 50/50 wt% from the surface principally consisting of HCPS; (**g**) PTMSP/HCPS 50/50 wt% from the surface principally consisting of PTMSP.

Next, to study the surface of the MMM, a measurement of the water contact angle was carried out. As can be seen from Figure 5, the contact angle for water for the initial hydrophobic PTMSP membrane was 90°. With the introduction of 10 wt% HCPS in PTMSP, the surface with a predominant content of HCPS exhibited a contact angle of 155°, which is characteristic of superhydrophobic materials (Figure 5b). On the reverse side of the hybrid membrane, the contact angle was similar to the PTMSP membrane without the addition of HCPS (Figure 5c). Thus, a so-called flat “Janus” membrane was obtained, having on the one hand a superhydrophobic surface (contact angle greater than 150°) and on the other hand a hydrophobic (90°) surface. The increase in the content of HCPS in the membrane to 30 wt% led to the increase in the water contact angle on both sides of the membrane (157° and 94°). With a content of HCPS of 50 wt%, the contact angle from the HCPS side reached 162°, and from the PTMSP side 94°. The increase in the contact angle is due to the resulting rough surface of the hybrid membrane during its casting. The study of the contact angle of the surface of the membrane, formed from PTMSP/HCPS 50/50 wt%, was also performed using BTX solution in water, with a total concentration of organic components of 0.03 wt%. The contact angle in that case was 147°. Thus, the introduction of hydrophobic HCPS nanoparticles into PTMSP leads to the creation of a membrane not only with an outstanding sorption capabilities to organic components, but also to the production of a “Janus” membrane, with a superhydrophobic rough surface on one side and a hydrophobic surface on the other side of the membrane.

### 3.2. Pervaporation Measurements

**Figure 6:**
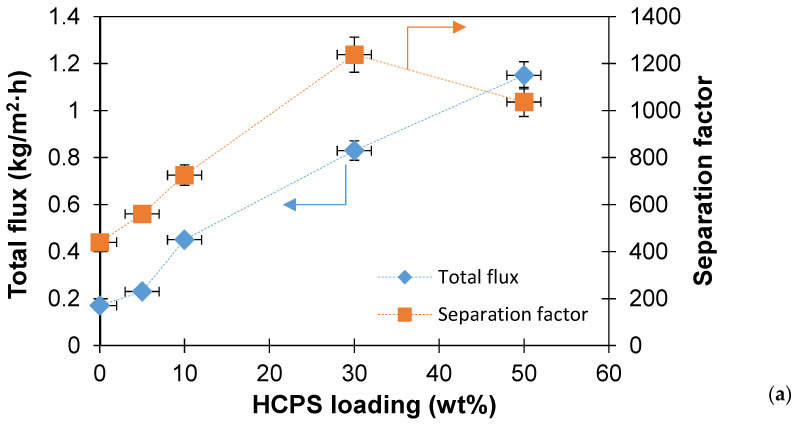

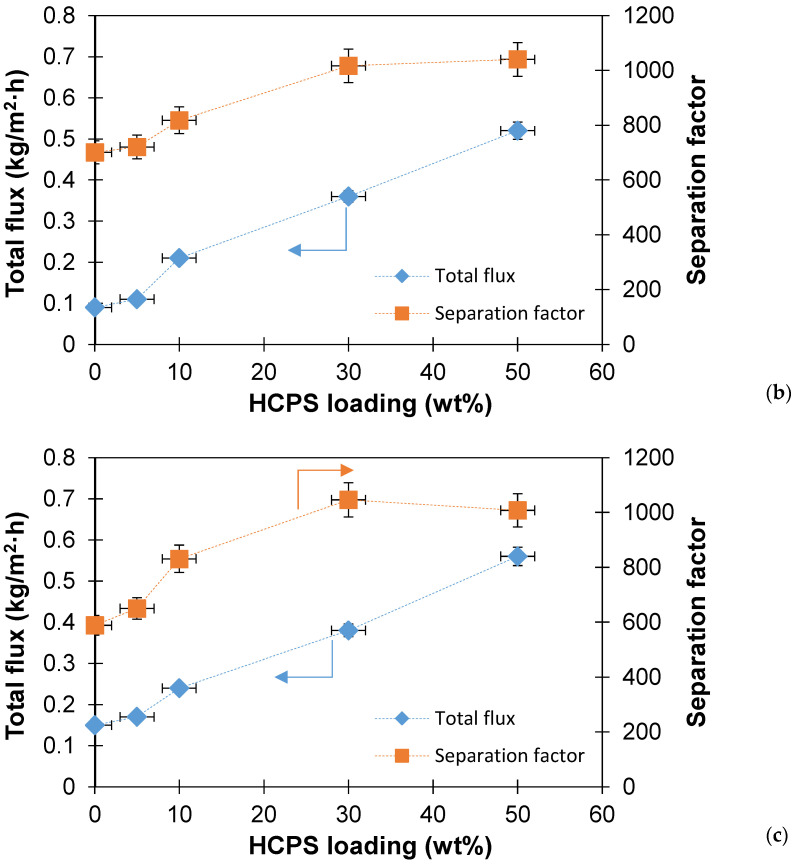
Dependence of permeate flux and separation factor on HCPS loading into PTMSP during the pervaporation separation of binary solutions benzene-water (**a**), toluene-water (**b**), o-xylene-water (**c**).

The initial PTMSP membrane and PTMSP membranes containing from 5 to 50 wt% HCPS were studied during the pervaporation separation of model binary solutions containing 0.15 wt% benzene in water, 0.05 wt% toluene in water and 0.016 wt% o-xylene in water. The concentrations of organic components in solutions were as close as possible to their solubility in water [47,48,49]. The initial experiments were carried out at the temperature of the separated solution of 30 °C. During the pervaporation experiments, the surface of the membrane with the predominant content of HCPS was in contact with the feed. The dependencies of the permeate flux and the separation factor from HCPS loading into PTMSP are shown in Figure 6. The permeate flux for all three binary solutions increased with HCPS loading into PTMSP. For example, compared with the initial PTMSP membrane, the permeate flux for the PTMSP/HCPS 50 wt% membrane was four times higher when separating o-xylene-water (0.15 vs. 0.56 kg/m^2^·h), six times higher when separating toluene-water (0.09 vs. 0.52 kg/m^2^·h) and seven times higher when separating benzene–water (0.17 vs. 1.15 kg/m^2^·h). This increase in permeate flux can be attributed to a significantly smaller dense layer of PTMSP in the case of PTMSP/HCPS membranes. According to the results of SEM, a solid layer of PTMSP in MMM, where the content of HCPS varied between 30–50 wt%, was 3–5 microns. The increase in the permeate flux in a row o-xylene < toluene < benzene is explained by the higher content of benzene in the separated solution and apparently better sorption, as well as swelling of the membrane material. Usually, with a decrease in the thickness of the membrane, the permeate flux increases, but the selectivity of separation decreases. Contrary to the expectations, PTMSP/HCPS membranes, obtained in the current work, showed a significant increase in the separation factor (1.5–2 times) in comparison with the original initial PTMSP membrane. The maximum separation factors (>1000) for all treated solutions were obtained for a PTMSP membrane with an HCPS content of 30 wt%. When HCPS loading increased to 50 wt%, the pollutant/water separation factor either remained at the same level or started to decrease. This dependence of the separation factor on the HCPS content in PTMSP can be explained either by achieving the optimum HCPS content (30 wt%), or by possible defects in the PTMSP dense layer for the PTMSP/HCPS 50 wt% membrane. Moreover, the same dependence was observed in the study of the repeatability of the pervaporative properties of hybrid membranes. Three membrane samples of the same composition were prepared in this work, and the difference in pervaporation experiments did not exceed 10%.

**Figure 7:**
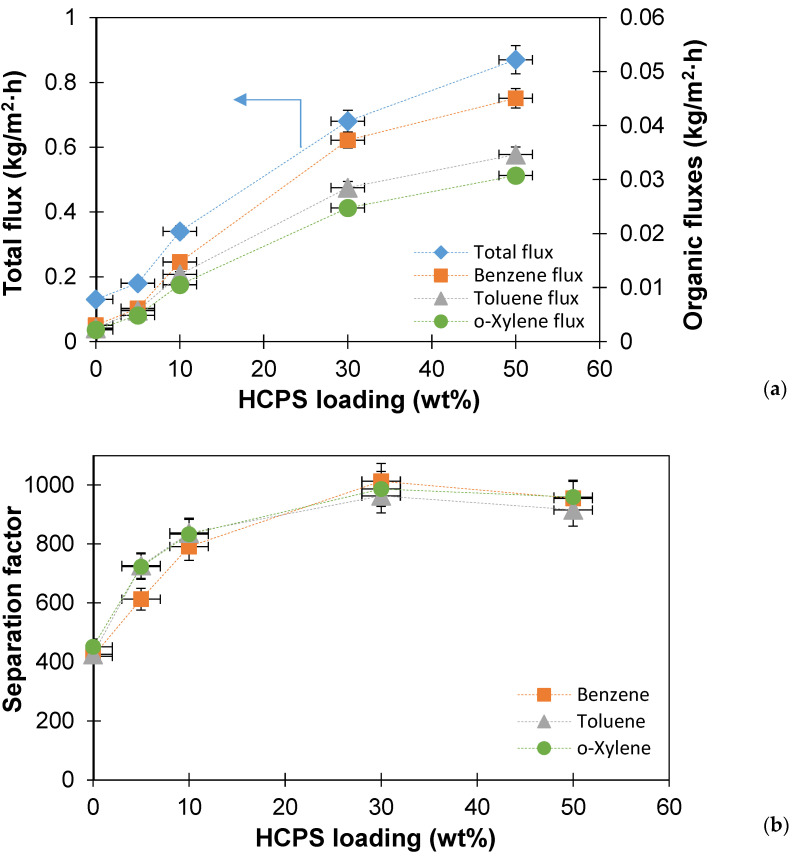
The dependence of the permeate flux, the flux of organic components (**a**) and the separation factor (**b**) on the HCPS loading into PTMSP during the pervaporation separation of a multicomponent BTX mixture.

The results of the pervaporation separation of multicomponent BTX mixture for PTMSP membranes with different HCPS loading are shown in Figure 7. The content of each of the organic components (benzene, toluene, o-xylene) in the solution was 0.01 wt%, thus, the total content of BTX was 0.03 wt%. As in the cases of separation of binary solutions, the permeate flux during the initial separation of a multicomponent BTX mixture increased 5.5 times with an increase in HCPS loading (Figure 7a) and for the PTMSP/HCPS 50 wt% membrane it reached 0.83 kg/m^2^·h. The increase in the flux of organic components with the addition of HCPS is more significant. It can be observed that the flux of benzene in comparison with the initial PTMSP membrane increased by 12 times with 50 wt% loading HCPS into PTMSP (0.0036 vs. 0.045 kg/m^2^·h). For each membrane, the fluxes of organic components increased in the following row: o-xylene < toluene < benzene (Figure 7a).

When BTX was removed from a multicomponent model solution, it was possible to increase the separation factor by more than two times for all organic components in comparison with the initial PTMSP membrane. This fact may indicate that HCPS, which has excellent sorbing properties, effectively sorbs organic components from an aqueous solution. As a result, the PTMSP/HCPS membrane swells, which increases the transport of target components through a thin layer of PTMSP, which also contains a small number of HCPS particles. Moreover, the separation factor for a multicomponent BTX solution, as in the case for binary solutions, has a maximum with an HCPS content of 30 wt% in PTMSP. At the same time, the organic/water separation factors for each membrane separately demonstrated quite close values.

A comparison of the pervaporation results obtained in this work and previously published in the literature is presented in Table 1. To compare the efficiency of membranes, the pervaporation separation index (PSI) is widely used in the literature, as it includes both the permeate flux and the separation factor. It can be seen that the PTMSP/HCPS membranes, obtained in this work, demonstrate better characteristics than the membranes presented in other sources during the pervaporation recovery of benzene, toluene and o-xylene from aqueous solutions. Thus, within the framework of this work, MMMs containing up to 50 wt% HCPS with high transport and separation characteristics were obtained, which is an important step towards the application of pervaporation as a promising method for removing volatile organic compounds from water.

## 4. Conclusions

In this work, for the first time, membranes based on poly(1-trimethylsilyl-1-propyne) (PTMSP) with a large load of HCPS sorbent particles (up to 50 wt%) were prepared and investigated for the vacuum pervaporation recovery of volatile organic compounds from water. The industrial sorbent Purolite Macronet™ MN200, with a high sorption capacity for organic solvents, was chosen as high free volume membrane filler. Using scanning electron microscopy, it was established that the membranes are asymmetric when HCPS content is higher than 30 wt%; the cross-section of membranes is similar to composite membranes (a clearly defined thin selective layer consisting mainly of PTMSP and a thick porous layer consisting mainly of HCPS). The selective layer is 3–5 µm thick and also contains HCPS particles; the support layer has a thickness of 50–60 µm. This porous layer consists of HCPS particles interconnected to each other by a PTMSP as a binding component. A study of the MMM surface was carried out by measuring the water contact angle. When HCPS was introduced into PTMSP, a surface with a predominant content of HCPS exhibited contact angle of more than 150°, which is characteristic of superhydrophobic materials. On the reverse side of the hybrid membrane, the contact angle was similar to the PTMSP material (90°).

The transport and separation characteristics of PTMSP membranes with different HCPS loading were studied in the pervaporation separation of binary solutions of benzene-water, toluene-water, o-xylene-water, and a multicomponent BTX-water mixture. The permeate flux increased significantly (by 4–7 times) as the content of HCPS in the composite increases. This increase in permeate flux is associated with a significantly lower thickness of the selective layer in the case of PTMSP/HCPS membranes. In addition to improving the transport properties, the PTMSP/HCPS membranes designed in this work, showed a significant increase in the separation factor (1.5–2 times) in comparison with the virgin PTMSP membrane. The maximum separation factors (>1000) for all treated solutions were obtained for the membrane with HCPS concentration of 30 wt%. When HCPS loading increased to 50 wt%, the organic component/water separation factor either remained at the same level or started to decrease. These novel PTMSP/HCPS membranes demonstrate higher organic flux during the pervaporation recovery of benzene, toluene and o-xylene from aqueous solutions than the membranes presented in other works.

It is expected that the PTMSP/HCPS membranes will effectively recover other pollutants from aqueous media, for example, phenols, which will open up new prospects for the application of the process, expand the range of separated systems, and lead to the development of the fundamental concepts of pervaporation.

## Figures and Tables

**Table 1:** Comparison of pervaporation performances.

Membranes	Conditions	Organic Component, wt%	Total Flux, kg/m^2^∙h	Separation Factor	PSI, kg/m^2^∙h	Ref.
MFI	30 °C	0.01 (Benzene)	0.12	64	7.6	[2]
		0.01 (Toluene)	0.25	53	13	
		0.01 (o-Xylene)	0.18	35	4.3	
PEBA	25 °C; 0.001 bar	0.02 (Toluene)	0.02	2450	49	[34]
PEBA with 15% carbon black	25 °C; 0.001 bar	0.02 (Toluene)	0.033	1800	59.4	[34]
PDMS	25 °C; 0.001 bar	0.02 (Toluene)	0.7	80	55.3	[34]
PDMS with 15% carbon black	25 °C; 0.001 bar	0.02 (Toluene)	0.3	200	59.7	[34]
PEBA	25 °C; 0.001 bar	0.05 (Toluene)	0.03	1500	45	[51]
PEBA with 15% carbon black	25 °C; 0.001 bar	0.05 (Toluene)	0.049	920	45	[51]
Polyvinylidene fluoride hollow-fiber	25 °C; 0.025 bar	0.08 (Benzene)	0.26	250	65	[52]
Poly(ethylmethacrylate)-PDMS	40 °C; 1.3 × 10^−5^ bar	0.06 (Benzene)	0.018	2400	43.2	[53]
Poly(methylmethacrylate)-PDMS	40 °C; 1.3 × 10^−5^ bar	0.05 (Benzene)	0.017	3700	63	[53]
PDMS	60 °C; 0.01 bar	0.14 (Benzene)	0.15	3302	495.2	[54]
PDMS with 3% tert-butylcalix [4] arene (CA)	60 °C; 0.01 bar	0.14 (Benzene)	0.13	5604	728.4	[54]
Polyether-block-amide (PEBA)	0.01 bar	0.03 (Toluene)	0.085	60	5	[55]
PEBA with 2% NaX nanozeolite	0.01 bar	0.03 (Toluene)	0.11	60	6.5	[55]
PTMSP	30 °C, 5 × 10^−5^ bar	0.15 (Benzene)	0.17	440	74.6	This work
		0.05 (Toluene)	0.09	701	63	
		0.016 (o-Xylene)	0.15	588	88	
PTMSP/HCPS 5 wt%	30 °C, 5 × 10^−5^ bar	0.15 (Benzene)	0.23	560	128.6	This work
		0.05 (Toluene)	0.11	721	79.2	
		0.016 (o-Xylene)	0.17	650	110.3	
PTMSP/HCPS 10 wt%	30 °C, 5 × 10^−5^ bar	0.15 (Benzene)	0.45	725	325.8	This work
		0.05 (Toluene)	0.21	818	171.6	
		0.016 (o-Xylene)	0.24	831	199.2	
PTMSP/HCPS 30 wt%	30 °C, 5 × 10^−5^ bar	0.15 (Benzene)	0.83	1238	1027	This work
		0.05 (Toluene)	0.36	1017	365.8	
		0.016 (o-Xylene)	0.38	1046	397.1	
PTMSP/HCPS 50 wt%	30 °C, 5 × 10^−5^ bar	0.15 (Benzene)	1.15	1037	1191	This work
		0.05 (Toluene)	0.52	1040	540.3	
		0.016 (o-Xylene)	0.56	1008	563.9	

## Data Availability

Not applicable.
